# The Influence of Participation in Pregnancy Courses and Breastfeeding Support Groups on Attitudes and Knowledge of Health Professionals about Breastfeeding

**DOI:** 10.3390/children10040632

**Published:** 2023-03-28

**Authors:** Marija Čatipović, Zrinka Puharić

**Affiliations:** 1 Department of Nursing, Bjelovar University of Applied Sciences, 43000 Bjelovar, Croatia; 2 Faculty of Dental Medicine and Health, Josip Juraj Strossmayer University of Osijek, 31000 Osijek, Croatia

**Keywords:** breastfeeding support groups, pregnancy courses, health professionals

## Abstract

Numerous factors affect the behavior, attitudes, and knowledge of health professionals about breastfeeding. The aim of this paper is to determine the impact of participation in pregnancy courses and breastfeeding support groups on the attitudes and knowledge of health professionals about breastfeeding. The study compares two groups of health professionals according to the results they achieved on a validated questionnaire of behavior, attitudes, and knowledge about breastfeeding. The authors did not make personal contact with the respondents, as the questionnaires were filled out online. The two groups of respondents differed according to the frequency of participation in pregnancy courses, that is, groups for breastfeeding support. The results are presented tabularly and graphically (frequencies and percentages), while differences in the results between the infrequent and regular participants are shown with the Mann–Whitney U test (asymmetric distribution). Better results on the questionnaire were achieved by those who regularly attended breastfeeding support groups (Mdn = 149, IQR = 11) in comparison to infrequent visitors (Mdn = 137, IQR = 23). The same is found for regular visitors of pregnancy courses (Mdn = 149, IQR = 15.75) in comparison to infrequent visitors (Mdn = 137, IQR = 23). The differences are statistically significant (*p* < 0.00). Partial correlation confirms a more significant influence of breastfeeding support groups (<0.00) than pregnancy courses (*p* = 0.34). Working in breastfeeding support groups had a statistically significant positive effect on the attitudes and knowledge of health professionals about breastfeeding. The topic of breastfeeding should be given more space and importance during pregnancy courses as well. Personal experience working in breastfeeding support groups and pregnancy courses should be incorporated into the training of medical students.

## 1. Introduction

Leading health organizations recommend exclusive breastfeeding for the first 6 months of the child’s life. Total breastfeeding is recommended for up to 24 months (or longer) because of the properties that breast milk has, which make it optimal for the child’s healthy development [1].

While discussing the reasons for unsatisfactory early initiation of breastfeeding, the duration of exclusive and total breastfeeding [2], both in the world and Croatia [3,4], the responsibility of health professionals should be considered. The Surgeon General of the United States said that some of the biggest obstacles to successful breastfeeding are inadequate education and training of health professionals. The Surgeon General of the United States recommended continuous training and education of health professionals, as well as periodic licensing and certification of health professionals, in order to ensure the quality of breastfeeding support [5]. All healthcare staff should be trained to implement the best breastfeeding policies [6].

The question is whether or not education and training programs for healthcare staff have an effect on their knowledge and attitudes about supporting breastfeeding women. Education should integrate embodied, substitute, practical and theoretical knowledge [7]. The above cannot be achieved without personal experience in clinical practice [8]. Depending on the organization, pregnancy courses and breastfeeding support groups can become an important part of the training program for future health workers. In Croatia, there is an education program for leaders of breastfeeding support groups and pregnancy course leaders supported by the Ministry of Health, the Croatian UNICEF Office, and the Croatian Association of Breastfeeding Support Groups. The breastfeeding support groups meet continuously two times a month. Mothers come to the groups for as long as they are breastfeeding. Most of the time, they come to group meetings with their children, often accompanied by their partners. The groups are semi-open; when one mother leaves, a new mother joins the group. On average, the groups consist of 8 mothers. In Croatia, the pregnancy course program takes place over five days. On the first day, a gynecologist talks about pregnancy, a police representative talks about the safe use of car seats, and a psychologist conducts psychological preparation for childbirth and parenthood. On the second day, a lawyer informs the parents about the rights that pregnant women have. On the third day, a pediatrician talks about the newborn, and the pediatric visiting nurses talk about the mother’s preparation for going to the maternity ward and taking care of the newborn. On the fourth day, a gynecologist talks about childbirth, and pediatric visiting nurses talk about breastfeeding and the period of midwifery. On the fifth day, a dentist talks about protecting a pregnant woman’s teeth, and a physiotherapist conducts practical exercises with pregnant women. Participation in breastfeeding support groups is free.

The second question is about measuring the effectiveness of the applied educational program. Two procedures are most often used as outcome measures: the breastfeeding rate and changes in participants’ knowledge, attitudes, and skills. Given that there are many other factors that influence breastfeeding rates, we believe that focusing on changes in behavior, knowledge and attitudes is a better choice [8]. To measure the changes in behavior, attitudes, and knowledge of health professionals about breastfeeding, validated questionnaires adapted to cultural, economic, social, religious, and other specifics of the concrete environment [9].

The aim of this paper is to determine whether or not healthcare professionals who have experience working in pregnancy courses/breastfeeding support groups differ in attitudes and knowledge from healthcare professionals who don’t have such experience. 

## 2. Materials and Methods

### 2.1. Study Design

This cross-sectional study compares the results of two groups of health professionals (from the Republic of Croatia) on breastfeeding behavior, attitudes, and knowledge questionnaire (BBAKQ prof.). The observed groups differ depending on their participation in the work of breastfeeding support groups and pregnancy courses. The authors expect better results in the group of respondents with experience in pregnancy courses and breastfeeding support groups.

### 2.2. Ethical Principles

The study was conducted according to the guidelines of the Declaration of Helsinki and approved by the Ethics Committee of the Bjelovar University of Applied Sciences. (date: 11 March 2021, number: 2103/01-21-01-21-02). 

The respondents are 374 health professionals (37 male and 337 female) from the Republic of Croatia. They were asked about the frequency of their participation in pregnancy courses. Respondents who answered “never”, “very rarely, only a few times” (1 to 5 times during their previous experience), “more than a few times, but not often” (answers between “very rarely, only a few times” and “often”) to the question of participation in pregnancy courses, were classified as “infrequent participants”. Respondents who answered with “often” (at least four times a year) and “regularly” (at least once a month) were put in the group of “regular participants.” There is no gold standard for how many courses healthcare workers should attend to provide adequate support for patients. There were 318 respondents in the “infrequent participants” group and 56 respondents in the “regular participants” group. In the same way, the respondents were divided according to their participation in breastfeeding support groups. The group of “infrequent participants” had 323 respondents, and the group of “regular participants” had 51 respondents. The compared groups of respondents (“Infrequent” and “Regular”) do not differ in sociodemographic characteristics.

### 2.3. Measuring Instrument

The Breastfeeding Behavior Attitudes and Knowledge Questionnaire for health professionals (BBAKQ prof.) was used in the research (Supplementary Materials Table S1). It has been validated and used in Croatia since 2020. The questionnaire consists of a behavior scale (9 items), an attitude scale (19 items) and a knowledge scale (20 items). Cronbach’s alpha (as reliability coefficient) for the behavior scale is 0.70, for the attitudes scale 0.94, and Kuder-Richardson 20 for the knowledge scale is 0.81. An exploratory factor analysis was performed for the behavior scale (Kaiser-Meyer-Olkin Measure of Sampling Adequacy = 0.697, Bartlett’s Test of Sphericity *p* < 0.000, Initial Eigenvalues Cumulative for three components = 66.14%) and the attitude scale (Kaiser-Meyer- Olkin Measure of Sampling Adequacy = 0.932, Bartlett’s Test of Sphericity *p* < 0.000, Initial Eigenvalues Cumulative for two components = 61.65%). A confirmatory factor analysis was performed for the behavior scale (Average variance extracted = 0.52; 0.52; 0.53, Average Shared Variance = 0.28, Maximum Shared Variance = 0.25, the square root of Average variance extracted latent constructs is greater than the correlation between any pair of latent constructs, HTMT = 0.55) and the attitude scale (Average variance extracted = 0.50; 0.52, Average Shared Variance = 0.18, Maximum Shared Variance = 0.18, the square root of Average variance extracted latent constructs is greater than the correlation between any pair of latent constructs, HTMT = 0.63). The questionnaire is available at the link listed in the references section of this paper [10]. The author of the questionnaire is willing to provide instructions for scoring the results of the questionnaire upon request, which should be sent to her e-mail address.

### 2.4. Data Collection Process

The questionnaire was posted on the websites of Josip Juraj Strossmayer University in Osijek and Bjelovar University of Applied Sciences. Healthcare professionals received information about the study and link to access the questionnaire from several sources: from the association “For a healthy and happy childhood”, direct contacts from colleagues who actively participate in breastfeeding promotional activities, through the Croatian Breastfeeding Support Association with the assistance of lecturers from Josip Juraj Strossmayer University in Osijek, Bjelovar University, and through UNICEF’s “Specialist Pediatric Clinics-Friends of Breastfeeding” [11]. All healthcare workers could participate in the research, regardless of their age, place of residence, workplace, professional qualification, etc. The work was primarily aimed at collecting quantitative data because the authors did not have personal contact with the respondents. People who filled out the online questionnaire had the opportunity to add their comments to the questionnaire. The comments were not processed because there were only a few, and they were mainly aimed at supporting the research or suggesting questions on which the questionnaire could be expanded.

### 2.5. Statistical Procedures

The calculator on the website “Calculator.net” [12] was used to calculate the sample size. The total number of health workers in Croatia is 50.179. The margin of error is 5%, and the Confidence level is 95%. Population proportion represented the percentage of respondents with a satisfactory level of knowledge and positive attitudes about breastfeeding (80%), and it was determined according to the results of a previous study published in “Children” [11]. The minimum number of necessary samples to meet the desired statistical constraints is 245.

Flatness and curvature of the BIAKQ questionnaire result on graphic displays for both groups of respondents (in relation to their participation in pregnancy courses/breastfeeding support groups) were asymmetric. Therefore, nonparametric procedures were used while analyzing the significance of differences in the observed groups. The correlation/partial correlation between the frequency of participation in pregnancy courses and breastfeeding support groups and the results of the BIAKQ questionnaire was calculated, along with the variables of age. A significance level of 0.05 was assigned, and all *p* values are two-sided. SPSS Statistics V22.0 was used in the analysis.

## 3. Results

The sociodemographic data are presented in Table 1.

From Table 1, we see that the compared groups of respondents (“Infrequent” and “Regular”) do not differ in sociodemographic characteristics. Over the years, course attendance has been increasing.

**Figure 1:**
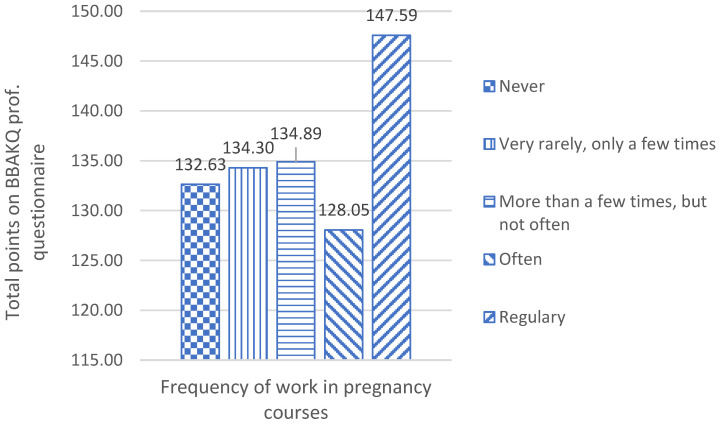
Arithmetic means of total points on BBAKQ prof. questionnaire according to the frequency of participation in the pregnancy courses (N = 374).

Figure 1 shows the results of respondents on the BBAKQ questionnaire in relation to their participation in the pregnancy courses. Total results increase proportionally to the frequency of participation in pregnancy courses, except in the “often” group. A more detailed analysis shows that the respondents’ results on the scale of attitudes are responsible for this result: M_Never_ = 83.83, SD = 13.43; M_Very rarely, only a few times_ = 84.00, SD = 13.34; M_More than a few times, but not often_ = 82.04, SD = 17.26; M_Often_ = 74.89, SD = 21.89; M_Regularly_ = 88.38; 11.70.

Only 9.9% of respondents regularly participated in pregnancy courses, 5.1% often participated, 7.5% more than a few times but not often, 16.3% very rarely-only a few times, and 61.2% of respondents never participated (Table 2). In the overall results, respondents who regularly participate in pregnancy courses had a better result (Mdn = 149.00) than those in the group of infrequent participation (Mdn = 137.00); the difference is statistically significant, U (N Infrequent = 318, N Regular= 56) = 5383.00, z = −4.721, *p* < 0.000). Only on the scale of attitudes do we not note a statistically significant difference in the results between the observed groups of respondents (“Infrequent” and “Regular”).

**Figure 2:**
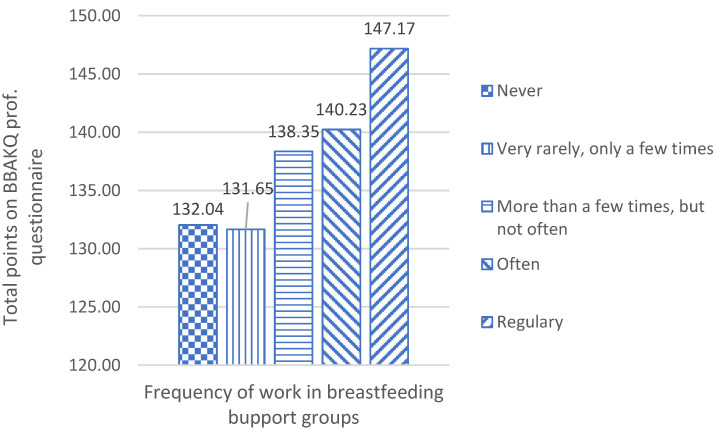
Arithmetic means of total points on the BBAKQ prof. questionnaire according to the frequency of participation in the breastfeeding support groups (N = 374).

Figure 2 shows the results of respondents on the BBAKQ questionnaire in relation to their participation in the breastfeeding support groups. Unsurprisingly the overall scores are higher when it comes to the more frequent attendees, essentially demonstrating that better education leads to better results.

Furthermore, 62.6% of the respondents never participated in the work of breastfeeding support groups, 13.1% participated very rarely, 10.7% participated more than a few times but not often, 5.9% participated often and 7.8% regularly (Table 3). In the overall results, respondents who regularly participate in the work of breastfeeding support groups had a better result (Mdn = 149.00) than those in the group of “infrequent participation” (Mdn = 137.00), and the difference is statistically significant, U (N Infrequent = 323, N Regular = 51) = 4293.00, z = −5.498, *p* < 0.000). On all scales of the questionnaire, we note a statistically significant difference in the results between the observed groups of respondents (“Infrequent” and “Regular”).

The correlation between participation in pregnancy courses and breastfeeding support groups is moderately strong. The correlation between participation in pregnancy courses/breastfeeding support groups and the respondents’ results on the questionnaire is weak but significant. The correlation between age and the results on the BBAKQ prof questionnaire is very weak (Table 4).

When we control the impact of breastfeeding support groups, the influence of frequency of participation in pregnancy courses loses significance in relation to the results of the questionnaire.

When we control the impact of pregnancy courses on the outcome, we still find a significant correlation between the frequency of participation in breastfeeding support groups and the respondents’ results on the questionnaire. 

When age is designated as a control variable, a statistically significant positive correlation with the results on the BBAKQ prof. questionnaire retains the variable of participation in breastfeeding support groups [r (371) = 0.20, *p* = *p* < 0.00] and the variable of participation in pregnancy courses [r (371) = 0.13, *p* = 0.01]. This excludes the responsibility of the variable of age for the previously mentioned result (significant correlation between the frequency of participation in breastfeeding support groups and the respondents’ results on the questionnaire when the variable of participation in pregnancy courses is controlled).

## 4. Discussion

More effort and time should be devoted to educating health professionals on breastfeeding, not only in the theoretical part but in the field of mastering practical skills as well [13]. Creating multidisciplinary teams that will be dedicated to the mother-child health relationship is needed [14]. The results of this research indicate a significant influence on participating in breastfeeding support groups (Table 2) and pregnancy courses (Table 3). Still, a small percentage of health professionals participate in pregnancy courses and breastfeeding support groups. Various training programs for healthcare professionals are designed and implemented [15], but they rarely ever include pregnancy courses and breastfeeding support group work [16]. In addition, the ways of organizing and operating pregnancy courses and breastfeeding support groups are uneven, which results in conflicting conclusions about their effectiveness. In some research, the effectiveness of breastfeeding support groups on breastfeeding rates is similar to the one of breastfeeding home visits. [17]. Other studies confirm that newborns whose mothers attended antenatal breastfeeding courses were almost twice as likely to achieve exclusive breastfeeding as recommended by UNICEF/WHO [18]. Croatia has very positive experiences with the influence of pregnancy courses and breastfeeding support groups on the initiation and duration of breastfeeding [19,20]. According to some authors, breastfeeding success does not correlate with participating in breastfeeding support groups but with the mother’s satisfaction, which is achieved in the group and according to the group leader [20].

Regular associates of breastfeeding support groups achieved better results on the behavior, attitudes, and knowledge questionnaire on breastfeeding than infrequent participants (Figure 2, Table 3). The breastfeeding support groups in Croatia are organized in such a way that the groups are led by health workers. In UNICEF’s “Specialist Pediatric Clinics-Friends of Breastfeeding” [21], the groups are led by pediatricians. In addition to those, there are also groups led by nurses or visiting nurses [22]. Less common are groups led by mothers who have completed breastfeeding support group leader training [23,24]. Most breastfeeding support groups are members of the Croatian breastfeeding support group association [25]. High school students at secondary medical school and medical college students are required to spend a part of their practice in primary health care. However, participation in breastfeeding support groups is not mandatory for them. They will only gain experience in breastfeeding support groups if the policlinic where they perform the practice participates in such activities.

Most women make the decision to breastfeed in the first trimester of their pregnancy or even before the pregnancy. Only a few women make this decision at the end of their pregnancy. In any case, most women make the decision to breastfeed before giving birth [26,27]. It has been found that the duration of breastfeeding is significantly related to the mother’s experience in pregnancy courses [19]. Therefore, it is important to emphasize that during the course, a physiotherapist, who is specialized in working with pregnant women, conducts practical exercises and preparations for childbirth with them [28]. Finally, even though students are required to spend a part of their practice in primary health care, whether or not they visit breastfeeding support groups/pregnancy courses during that practice depends on the doctor they are with. The results of the BBAKQ prof questionnaire show that regular participants of pregnancy courses have statistically significantly better results in comparison to irregular participants (Figure 1, Table 2). Accordingly, it is desirable for students to follow at least one pregnancy course from beginning to end and to make 2 to 3 visits to breastfeeding support groups.

The partial correlations analysis (Table 5—the control variable is participating in the breastfeeding support group) didn’t show significantly better results for participating in pregnancy courses or age. The analysis of partial correlations (Table 6—the control variable is participating in pregnancy courses) shows significantly better results for participating in breastfeeding support courses and not for age. A more significant impact of participating in breastfeeding support groups compared to pregnancy courses is understandable because breastfeeding topics are more represented in breastfeeding support group programs. In the program pregnancy courses, a short period of one hour is dedicated to the topic of breastfeeding. During breastfeeding support groups, breastfeeding is the central topic. The authors believe that this should be changed and that the topic of breastfeeding should be given more space and importance in pregnancy courses as well. It is useful to notice the increase in course attendance in proportion to age, which could mean that, with the acquisition of personal and professional experience, the awareness of breastfeeding importance also increases.

Healthcare professionals are more effective in supporting breastfeeding if they have positive personal attitudes about breastfeeding and if they possess practical skills in helping breastfeeding mothers. Formal education does not provide the necessary knowledge and skills to health professionals in this area. The hesitancy and the lack of knowledge and skills of health professionals result in less professional or non-professional people advising mothers on breastfeeding [29]. Research on the effectiveness of breastfeeding education conducted on a sample of healthcare students confirms that the only true effectiveness is one of well-structured breastfeeding education programs that include practical work and practical exercises and experience [30]. Other authors also suggest systematic changes in medical teaching and clinical practice. For example, they emphasize that medical schools are not adequately preparing students for supporting breastfeeding patients [31]. It is also emphasized that educational goals for medical schools and specialized training should be more focused on education and training with the aim of becoming familiar with the basics of breastfeeding and acquiring the ability to solve clinical problems related to breastfeeding [32]. However, very rarely do these new educational goals, aimed at improving breastfeeding knowledge and skills of future healthcare workers, resonate with the personal experience of working in breastfeeding support groups. The authors of this paper suggest that personal experience of working in breastfeeding support groups should be implemented in the education of medical students.

As far back as 1998, Humenick warned of the problem of inconsistency in the interventions of healthcare professionals in support of breastfeeding and the consequences that result from this [33]. A quarter of a century later, this problem should not exist. We all know that health professionals, with supportive attitudes and practical skills, significantly influence a mother’s decision to start breastfeeding and persist in breastfeeding. However, it is not quite that simple. Duarte warns that the knowledge of health professionals about breastfeeding is conflicting and that there is no research on the knowledge of dentists [34]. Pharmacists often make unnecessary recommendations about stopping breastfeeding while taking medication [35]. Turner states that during the COVID pandemic, health workers in several countries did not always act in accordance with the recommendations of the World Health Organization on breastfeeding [36]. Almeida concludes that breastfeeding is a challenge for health professionals, regardless of their specialization [14]. Radzyminski talks about the significant lack of skill in the assessment and management of breastfeeding couples [37]. Shattnawi warns of the contradiction that exists between the staff’s beliefs and behavior in relation to breastfeeding and supporting mothers [38]. This refers to the problem of attitudes, knowledge, and skills in providing breastfeeding support. We conclude, like Dykes, that it is time for an effective integration of embodied, practice-based and theoretically confirmed knowledge into the daily work of healthcare professionals [7]. We consider that negative attitudes of healthcare workers about breastfeeding are a significant obstacle to progress. We also noticed that the difference between the examined groups was the smallest on the scale of attitudes. The cognitive component of attitude consists of knowledge and beliefs; the conative component consists of the intention (tendency to do something), which means that the attitude will be realized through behavior, at least in one part, knowingly and willingly. However, attitude resistance to change is often associated with an underlying latent construct. Attitudes to which people attach more personal importance are more resistant to change. The emotional component gives attitudes persistence, firmness, and motivation dimension. Attitudes have a defensive function. Change that threatens the self will be difficult to realize. By including commitment in the rational choice model, the link between choice and personal well-being is extended to intangible gain, where people’s choices do not always consider their personal goals alone (e.g., when we donate to charity) [39]. In our case, this means that healthcare workers whose children are not breastfed may feel guilty when faced with information about the benefits of breastfeeding. Guilt can be the cause of unconscious resistance to positive attitudes about breastfeeding. The theoretical solution is in what has already been said, which is that behavior does not have to be motivated exclusively by immediate self-interest but also by the welfare that we achieve through sympathy and commitment. In other words, even health professionals who have not breastfed their children can develop positive behaviors and attitudes towards breastfeeding and helping other parents, but this is a process that requires effort and time. Unfortunately, it was not possible to confirm this theoretical assumption in the current research and additional data is needed, which should be obtained through qualitative-quantitative procedures.

The advantage of this study is the simple, practical application of results. It does not take a lot of effort to include the experience of working in breastfeeding support groups and pregnancy courses in the practical training of future health workers and updating the knowledge and skills of employed health workers. The weakness of this study is the relatively small sample of respondents from a limited geographical, cultural, and linguistic area. The results of this research are influenced by the way breastfeeding support groups are organized. As the paper points out, in Croatia, leaders of breastfeeding support groups are health workers. In environments where the work of breastfeeding support groups is organized differently than described in this paper, different research results are possible. These weaknesses can be overcome by organizing online research that will include a larger number of respondents from different countries, which can be realized by using social networks that bring health workers together.

This study revealed another problem. We could not answer the question of why the vast majority of healthcare personnel did not attend training and which educational approaches would be attractive to healthcare professionals because the authors did not have direct contact with the respondents. In order to get the answers to these questions, we intend to supplement the next research with qualitative methods, e.g., interviews, supervision, etc. There is also a lack of knowledge on healthcare workers with better results on the BBAKQ questionnaire having better results in practice, as well as, i.e., that their patients achieve better breastfeeding results compared to others. In order to find this out, it is necessary to conduct a follow-up qualitative-quantitative research lasting for at least two years.

## 5. Conclusions

It is important to note that the education of health professionals on breastfeeding is not only focused on knowledge but also on attitudes and counseling skills, as well as supporting breastfeeding mothers. An effective method of positively changing the attitudes and skills of breastfeeding support is to engage (future and current) health workers in the work of breastfeeding support groups. The results of this research indicate the need for greater representation of breastfeeding in pregnancy courses.

## Figures and Tables

**Table 1:** The demographics table for the respondents (N = 374).

		Participation in Pregnancy Courses		Participation in Breastfeeding Support Groups	
GENDER		Infrequent	Regular	Infrequent	Regular
Male	N	33	4	33	4
	%	8.82	1.07	8.82	1.07
Female	*n*	285	52	290	47
	%	76.20	13.90	77.54	12.57
TOWN		Infrequent	Regular	Infrequent	Regular
Zabok	*n*	81	9	88	2
	%	21.66	2.41	23.53	0.53
Osijek	*n*	72	8	66	14
	%	19.25	2.14	17.65	3.74
Zagreb	*n*	50	9	51	8
	%	13.37	2.41	13.64	2.14
Slavonski Brod	*n*	26	5	27	4
	%	6.95	1.34	7.22	1.07
Bjelovar	*n*	7	2	7	2
	%	1.87	0.53	1.87	0.53
Dubrovnik	*n*	4	0	4	0
	%	1.07	0.00	1.07	0.00
Karlovac	*n*	4	0	4	0
	%	1.07	0.00	1.07	0.00
Other cities	*n*	74	23	76	21
	%	19.79	6.15	20.32	5.61
PROFESSION		Infrequent	Regular	Infrequent	Regular
Pharmacy technician	*n*	2	0	2	0
	%	0.53	0.00	0.53	0.00
Physiotherapist	*n*	1	0	1	0
	%	0.27	0.00	0.27	0.00
Physiotherapist bachelor’s degree	*n*	3	1	4	0
	%	0.80	0.27	1.07	0.00
Doctor	*n*	11	7	14	4
	%	2.94	1.87	3.74	1.07
Nurse	*n*	93	3	93	3
	%	24.87	0.80	24.87	0.80
Nurse bachelor’s degree	*n*	170	37	164	40
	%	45.45	9.89	43.85	10.70
Midwife	*n*	29	0	28	1
	%	7.75	0.00	7.49	0.27
Midwife bachelor’s degree	*n*	7	6	14	2
	%	1.87	1.60	3.74	0.53
Other professions in healthcare	*n*	2	2	3	1
	%	0.53	0.53	0.80	0.27
AGE		Infrequent	Regular	Infrequent	Regular
	M	36.93	42.14	37.21	40.88
	SD	10.25	11.72	10.15	12.94

**Table 2:** Overview of Mann-Whitney U test results for respondents’ scores on the BBAKQ prof. questionnaire depending on participation in pregnancy courses (N = 374).

The Dependent Variable	Participation in Pregnancy Courses	N	Median	Interquartile Range	Mean Rank	Sum of Ranks	Mann-Whitney U	*p*-Value
Total points on BBAKQ prof. questionnaire	Infrequent	318	137	23	176.43	56,104.00	5383.00	<0.00
	Regular	56	149	15.75	250.38	14,021.00		
Points on behavior scale BBAKQ prof. questionnaire	Infrequent	318	33	9	171.79	54,630.00	3909.00	<0.00
	Regular	56	40	5.75	276.69	15,495.00		
Points on attitudes scale BBAKQ prof. questionnaire	Infrequent	318	89	13.25	184.64	58,715.00	7994.00	0.22
	Regular	56	91	8	203.75	11,410.00		
Points on knowledge scale BBAKQ prof. questionnaire	Infrequent	318	18	3	175.51	55,811.00	5090.00	<0.00
	Regular	56	19	2	255.61	14,314.00		

**Table 3:** Overview of Mann-Whitney U test results for respondents’ scores on the BBAKQ prof. questionnaire depending on their participation in breastfeeding support groups (N = 374).

The Dependent Variable	Participation in Breastfeeding Support Groups	N	Median	Interquartile Range	Mean Rank	Sum of Ranks	Mann-Whitney U	*p*-Value
Total points on the BBAKQ prof. questionnaire	Infrequent	323	137	23	175.29	56,619.00	4293.00	<0.00
	Regular	51	149	11	264.82	13,506.00		
Points on the behavior scale of the BBAKQ prof. questionnaire	Infrequent	318	33	9	172.50	55,716.00	3390.00	<0.00
	Regular	56	40	5	282.53	14,409.00		
Points on the attitudes scale of the BBAKQ prof. questionnaire	Infrequent	318	88	14	181.90	58,754.50	6428.50	0.01
	Regular	56	91	7	222.95	11,370.50		
Points on the knowledge scale of the BBAKQ prof. questionnaire	Infrequent	318	18	3	176.99	57,166.50	4840.50	<0.00
	Regular	56	19	2	254.09	12,958.50		

**Table 4:** An overview of the correlation of variables’ results on the BBAKQ prof. questionnaire, participation in the breastfeeding support groups/pregnancy courses and age (N = 374).

Variable	Statistics	Total Points on the BBAKQ Prof. Questi-Onnaire	Participation in Breasfeeing Suport Groups	Participation in Pregnancy Courses	Gender *
Participation in breasfeeding suport groups	Spearman’s rho	0.29			
	*p*-value	0.00			
Participation in pregnany courses	Spearman’s rho	0.24	0.51		
	*p*-value)	0.00	0.00		
Age	Spearman’s rho	0.11	0.07	0.15	−0.05
	*p*-value)	0.03	0.18	0.00	0.35

* The mark for female gender was 0, for male 1.

**Table 5:** Partial correlation for variables: results on the BBAKQ prof. questionnaire, participation in pregnancy courses and “age” (control variable = participation in breastfeeding support groups) (N = 374).

Variable	Statistics	Total Points on the BBAKQ Prof. Questi-Onnaire	Participation in Pregnancy Courses
Participation in pregnancy courses	Correlation Coefficient	0.05	
	Sig. (2-tailed)	0.34	
Age	Correlation Coefficient	0.08	0.13
	Sig. (2-tailed)	0.12	0.01

**Table 6:** Partial correlation of variables: results on the BBAKQ prof. questionnaire, participation in breastfeeding support groups and age (control variable = participation in pregnancy courses) (N = 374).

Variable	Statistics	Total Points on the BBAKQ Prof. Questi-Onnaire	Participation in Breasfeeding Suport Groups
Participation in breasfeeding suport groups	Correlation Coefficient	0.15	
	Sig. (2-tailed)	<0.00	
Age	Correlation Coefficient	0.08	0.03
	Sig. (2-tailed)	0.13	0.50

## Data Availability

The data presented in this study are available on request from the corresponding author. The authors do not have the consent of the participants for public distribution of the database.

## Supplementary Materials

The following supporting information can be downloaded at: https://www.mdpi.com/article/10.3390/children10040632/s1, Table S1: The Breastfeeding behavior attitudes and knowledge questionnaire for healthcare staff.
